# *In Vitro* Quantitative Evaluation of Postprocessing Filter for Metal Artifact Reduction in Cone Beam Computed Tomography Images of Titanium and Zirconium Dioxide Implants

**DOI:** 10.1155/2022/1362473

**Published:** 2022-02-24

**Authors:** Andre Luiz Ferreira Costa, Karolina A. C. Fardim, Jennifer M. Mantoani, Ana Lucia Franco Ricardo, Maria Aparecida N. Jardini, Kaan Orhan, Sérgio Lúcio Pereira de Castro Lopes

**Affiliations:** ^1^Postgraduate Program in Dentistry, Cruzeiro do Sul University (UNICSUL), São Paulo, SP, Brazil; ^2^Department of Diagnosis and Surgery, Science and Technology Institute, São Paulo State University, São José dos Campos, São Paulo, Brazil; ^3^Department of Dentomaxillofacial Radiology, Faculty of Dentistry, Ankara University, Ankara, Turkey

## Abstract

**Objective:**

To evaluate a postprocessing filter of a new imaging-processing software for analysis of metal artifact reduction.

**Methods:**

Eight artificial edentulous mandibles (phantoms), where titanium and zirconium dioxide implants had been installed in four different regions (i.e., incisors, canine, premolars, and molars). CBCT volume was acquired, and then, four types of filters were applied to the images: BAR filter and Multi-CDT NR filter (e-Vol DX) and Sharpening Filters 1x and 2x (OnDemand). Artifact was assessed by measuring the standard deviation (SD) of the gray values of filtered and unfiltered images. The comparison between implant material, teeth, and filters was performed by using ANOVA, whereas multiple comparisons were performed by using Bonferroni's test. The level of significance adopted was 5%.

**Results:**

The results showing higher SD values, which suggests a worse image, were obtained with titanium implants compared to zirconium dioxide ones. With regard to the four filters used, it can be seen that the lowest SD values were obtained with BAR and Multi-CDT NR filters and the highest with Sharpening Filters 1x and 2x, with no statistical difference between them, except regarding the molar region in titanium implants.

**Conclusion:**

The highest SD values were seen in zirconium dioxide implants, mainly in the region of anterior teeth. The BAR filter was found to be the most effective as its SD value decreased significantly, indicating that the image quality was improved.

## 1. Introduction

The growing demand for the use of cone beam computed tomography (CBCT) is now a routine in the field of implantology, not only for guiding the diagnosis and treatment planning but also for conducting postoperative evaluations when clinical examination and conventional radiography fail to provide sufficient diagnostic information [1–3]. However, when implants are present, the CBCT images are influenced by the high-density materials composing them. This generates the so-called artifacts, which interfere with the image quality and result in a doubtful diagnostic value, and this in turn may lead to false interpretations [1, 2, 4, 5] as the underlying structures are masked on the image.

Image artifacts are defined as being any distortion observed in reconstructed images and which are not related to the object under investigation [1, 2, 6]. The factors causing artifacts in CBCT images may be mainly associated with high-density materials and some device parameters, such as size of the FOV (field of view). Other parameters also change image quality, among them kilovoltage (kV), milliamperage (mA), and voxel size (element volume). Other factors such as device calibration and patient movements can also generate artifacts [6, 7].

Metal objects in patients can generate several physical effects on image quality as noise, beam hardening, scatter, and photo starvation [8–10]. An implant can generate beam hardening artifacts in which white and black lines are shown [11], making anatomical structures ambiguous and influencing the contrast between adjacent regions. Thus, these effects can seriously interfere with the diagnostic process by using CBCT [6].

In order to improve the image quality, some researchers have alternatively tested the use of metal artifact reduction (MAR) software despite the lack of consensus on the reliability of this tool [12–14]. When interpreting digital images (e.g., CBCT volume), the radiologists use resources available in the software to improve their quality in relation to the original images [12, 13, 15].

MAR requires an initial segmentation of metal objects in the originally reconstructed images before employing an algorithm to eliminate metal traces in the raw data and to reconstruct an image with minimization of artifacts [16] for a more accurate diagnosis, which contributes to an effective treatment planning [1, 17].

Each CBCT manufacturer markets a MAR algorithm offering tools for manipulating contrast and brightness as well as for reducing specifically artifacts [12, 17, 18] and visualizing structures hidden by them. A previous study, which evaluated the influence of MAR filters on CBCT images of titanium and zirconium implants, concluded that this tool should be activated when available as it has been shown to be effective in reducing artifacts [18]. However, this tool is activated at the time of image acquisition and not all CBCT machines have it available. Thus, only tools provided by MAR algorithms are available to improve images in cases where it is necessary to minimize the effects of artifacts on diagnosis.

Image postprocessing is a digital medical imaging technique in which the main objective is to modify an image to enhance diagnostic interpretation [19]. Postprocessing techniques involve the use of filters to enhance or suppress certain features of the image [19, 20] by decreasing noise, altering contrast, and changing the sharpness of the image [19, 21–23].

Some authors [22–24] have achieved positive results by employing postprocessing filters in order to minimize the presence of metal artifacts produced by obturation or intracanal posts in CBCT images.

To our knowledge, there is no study particularly investigating the performance of commercially available postprocessing filters to improve the visualization of images containing dental metal artifact in CBCT volumes.

The purpose of this study was to assess the performance of a new CBCT software called e-Vol DX (CDT Software, Bauru, SP, Brazil) for reducing metal artifacts in images of implants by using two postprocessing filters and by comparing them with two other filters (OnDemand, CyberMed Inc., Seoul, Korea). The null hypothesis was that there is no difference in the filter application regarding the quantification of artifacts.

## 2. Materials and Methods

### 2.1. Study Sample

This is an *in vitro* study which was approved by the Research Ethics Committee of the UNESP School of Dentistry according to protocol number 26758819400000077.

Eight edentulous mandibles [17] with alveoli made of barium were used as a phantom (Nacional Ossos, Jaú, SP, Brazil). The areas of teeth #41, #43, #44, and #46 were prepared to receive the implants. Titanium (SIN, São Paulo, SP, Brazil) and zirconium dioxide (Straumann®, AG, Switzerland) implants, external hexagon, measuring 3.75 mm in diameter × 13 mm in length were alternately inserted in the phantoms. Therefore, a total of 8 mandibles, each containing one implant in the single tooth gap, were used.

### 2.2. Image Acquisition

**Figure 1:**
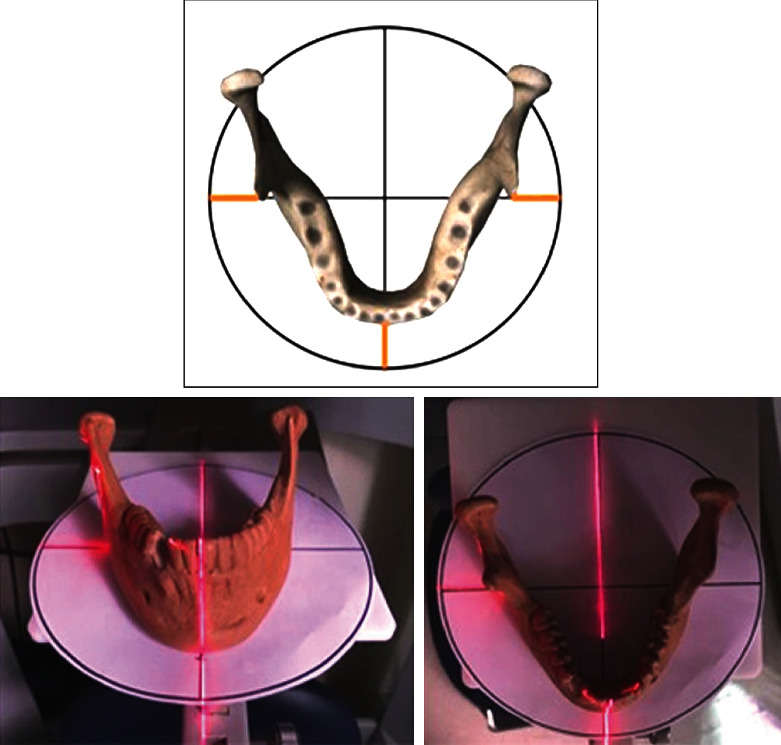
Schematic figure showing phantom positioned on the paper template with laser light markers.

Before acquiring the images, the phantom was attached to the machine's support plate and a paper template was placed on it. Next, a 16 cm diameter circle (corresponding to the FOV diameter used for image acquisition) was drawn on the paper template with four quadrants to represent the center of the FOV for positioning of the laser lights (Figure 1).

The phantoms were placed by centralizing the orientation template at the FOV so that the symphysis (midline) and the right and left lateral edges were equidistant from the circle's edges, thus making the laser beams intercept them. In this way, one could standardize the position of all phantoms as they had the same dimensions, which were obtained by using a millimeter ruler in relation to the center of the FOV.

Each implant was individually scanned and images acquired by using an I-Cat Next Generation unit (Imaging Sciences International, Hatfield, PA, USA) operating with the following parameters: 120 kV, 10 mA, FOV of 16 × 6 cm, and voxel of 0.20 mm. The CBCT images were acquired three times for each implant in order to reduce possible interference from variations during the scanning process. A total of 24 CBCT images were obtained. To keep the voxel size constant and avoid changes in image acquisition factors for prolonged use of the scanner, four images were acquired at a time each day, with an interval of 25 minutes between each acquisition, for monitoring the protocol calibration.

### 2.3. Image Analysis

#### 2.3.1. Image Selection

**Figure 2:**
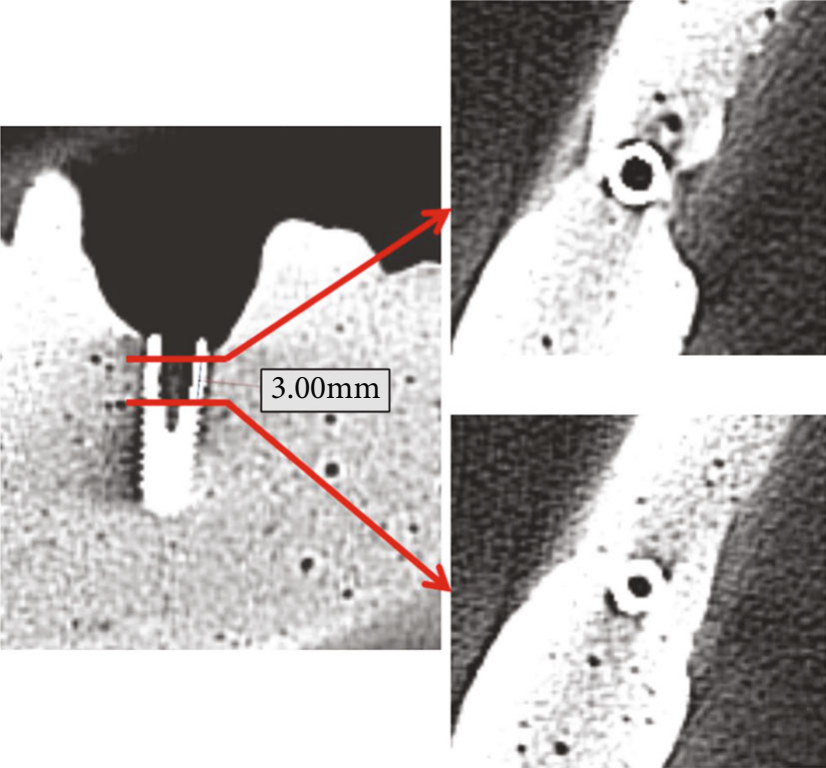
Selection of axial sections from CBCT images.

After acquisition of the images, which were in DICOM format (Digital Imaging and Communications in Medicine), the data were exported to the RadiAnt DICOM Viewer (Medixant, Poznan, Poland). Initially, the axial section of the implant's base was identified through numbered sections. The axial section at 3.0 mm from the apex of the implant was selected in the cervicoapical direction (also numbered), which encompassed the implant and the surrounding bone tissue, thus being the most representative section of the artifact in the cervical region of common cases of peri-implant bone loss (Figure 2).

#### 2.3.2. Image Filters

Since not all CBCT units available on the market have MAR algorithms, which enables the reduction of artifacts, some professionals have the only option to improve the quality of an image containing metal artifact by using own software enhancement resources. Therefore, we have decided to use two sharpening filters for image enhancement (OnDemand software) as well as unfiltered images (original images for comparison with those filtered by e-Vol DX software).

DICOM data sets were exported to both software, and then, the four types of filters were applied as described below.

### 2.4. e-Vol DX Software

The BAR (Blooming Artifact Reduction) filter, according to the manufacturer's guidelines, eliminates the effect of artifacts resulting from high-density materials (e.g., implants and intracanal metal posts), which prevents loss of information and allows an effective diagnosis to be made. The filter scans the dynamic range of the files and acts by normalizing of rows and columns to a grayscale value, thus preserving spatial resolution and removing bright streaks.

The Multi-CDT NR (noise reduction) filter, according to the manufacturer's guidelines, reduces the graininess of the CBCT images and allows better discrimination and sharpness of the details.

### 2.5. OnDemand Software

Filter 1x enhances the density transitions at a mild intensity.

Filter 2x enhances the density transitions at a high intensity.

### 2.6. Quantification of Artifacts

A previously calibrated dentomaxillofacial radiologist, with five years of experience in interpreting CBCT images, assessed all the images (DICOM format) on a 23.8-inch LCD monitor (Dell ultrasharp, wide screen flat-panel monitor) under dim light conditions. Calibration was carried out with images that were not included in the sample. Evaluations were carried out as follows: the examiner applied the filters of each software to 16 CBCT images separately, twice within an interval of one week and then the artifacts were quantified. Next, the results were submitted to intraclass correlation coefficient (ICC) test and the examiner was considered calibrated when an excellent coefficient was reached (ICC > 0.90).

By using the ImageJ software (National Institutes of Health, Maryland, MD, USA) with the circular tool, it was possible to create a ROI (region of interest) of 10.00 mm in diameter coinciding with the center of the implant. Subsequently, the region corresponding to the implant (i.e., 3.75 mm) was deleted from the image, resulting in a 6.25 mm circular crown to be analyzed to ensure quantification of that ROI not included on the image of the implant, which could change the result of the analysis.

The artifacts were quantified according to the methodology proposed by Pauwels et al. [25] and adapted by Machado et al. [14] as follows: once the ROI was selected, a histogram was generated by using the histogram analysis tool to determine the grayscale range and to obtain the minimum and maximum grayscale values, which were used to calculate the actual standard deviation (SD).

The CBCT unit used in this study generates images with a 16-bit scale (65.536 gray values), which allowed us to obtain the maximum SD, at least theoretically, corresponding to half of the gray values (32.768 gray values). The measurement was made by using the following formula: (actual SD/theoretical maximum SD) × 100. Analyses of the 16-bit images were performed, and the mean value was calculated from the three scans of each implant.

The dentomaxillofacial radiologist performed the measurements twice after a 15-day interval to estimate intrarater variability.

### 2.7. Statistical Analysis

Normality and homoscedasticity of the data were performed by using the Shapiro-Wilk and Bartlett tests, respectively. As the results indicated that data were normally distributed (parametric data), both data analysis and graph construction were performed. ANOVA was used for comparison between implants, teeth, and filters regarding their effects on artifact quantification and their interactions. The triple interaction between implants, teeth, and filter was significant (*P* value < 0.001), indicating that the artifact quantification behaves differently depending on the combinations between implants, teeth, and filters. Due to the significant triple interaction, it is not possible to interpret the effects of implants, teeth, and filters. In this case, a significant interaction between teeth and filter was observed in both titanium (*P* value < 0.001) and zirconium dioxide (*P* value < 0.001) implants, so ANOVA was performed for each implant to make it impossible to interpret the effects of teeth and filter. Finally, it was necessary to make models for implants and teeth to compare the filters with each other within each combination of implant and teeth. Multiple comparisons were also performed by using the Bonferroni test, and intrarater variability was analyzed by using the ICC test.

All statistical analyses were performed by using the R software, version 3.6.0 (The R Foundation for Statistical Computing), at a significance level of 5%.

## 3. Results

An excellent repeatability was found for intrarater reliability (ICC = 0.99), and the ANOVA model showed good power of prediction (*R*^2^ adjusted = 99.3%).

**Figure 3:**
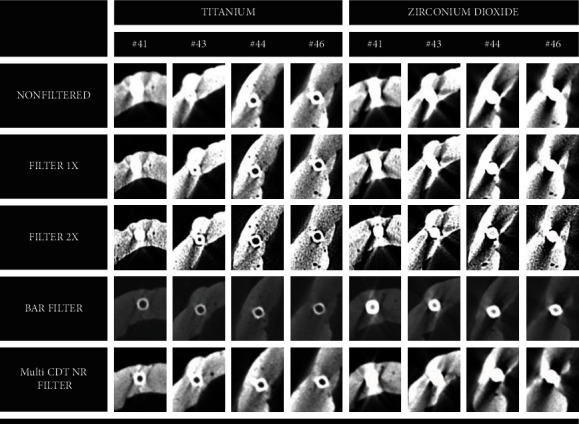
Example of artifacts generated by titanium and zirconium dioxide implants in images of teeth with application of the four filters and unfiltered images.

Figure 3 illustrates the CBCT images with different filters and artifacts generated by titanium and zirconium dioxide implants.

Table 1 shows the quantification of artifacts for implant material and tooth region, respectively, regarding the four filters and unfiltered images. It is important to note that higher values for artifact quantification are related to a worse filter performance (i.e., artifact reduction), whereas lower values are related to a better performance. It was found that zirconium dioxide implants had higher mean values than titanium ones, that is, worse results. For instance, although all values were very close to each other when Filter 1x was used, one can see that the mean artifact quantification for titanium implants in the regions of teeth #44 and #41 was greater than that in the region of tooth #43, which in turn is greater than that in the region of tooth #46. On the other hand, when the Multi-CDT NR filter was used, the mean quantification of artifact for titanium implant in the region of tooth #41 was greater than that in the region of tooth #43, which in turn was greater than that in the region of tooth #46, and which was also greater than that in the region of tooth #44. Moreover, all values were very different between each other. That is, the artifact quantification behaves differently depending on the tooth region and filter used.

The lowest mean values of quantification of artifact were observed in the region of tooth #46, except for titanium implants with BAR and Multi-CDT NR filters (both belonging to e-Vol DX software), whose mean values of quantification of artifact in the region of tooth #44 were lower than those found in the region of tooth #46. Nevertheless, the mean values of artifact quantification in the region of tooth #46 were the second lowest for these two filters. In the case of BAR filter, the mean values were very close to those found in the region of tooth #44, which were the lowest.

In the case of titanium implants, the highest mean values of quantification of artifact were found in the region of tooth #41, regardless of the filter used, except for BAR filter, whose mean values in the region of tooth #43 were slightly higher than those in the region of tooth #41, but very close to each other. For zirconium dioxide implants, the highest mean values of quantification of artifact also occurred in the region of tooth #41, except for filters of e-Vol DX software (BAR and Multi-CDT NR filters), whose mean values in the region of tooth #41 were the second lowest, being only higher than those in the region of tooth #46, as previously reported.

Considering the mean values of quantification of artifacts in unfiltered images and comparing them to the filtered ones, it can be seen that the mean values were decreased in both implants with the use of the four filters. In the case of titanium implants, it should be emphasized that the values related to posterior areas (i.e., regions of teeth #44 and #46) were slightly higher in images filtered with Filter 1x than in unfiltered ones. In the case of zirconium dioxide implants, the values were higher only in the region of tooth #44 in images filtered with Filter 1x.

To assess the effects of tooth region, implant material, and filters on the quantification of artifacts, ANOVA was performed with these three factors and their interactions. The triple interaction between implant material, tooth region, and filters was significant (*P* < 0.001), indicating that the quantification behaves differently depending on the combinations of these factors. Due to the significant triple interaction, it was not possible to interpret the effects of implant material, tooth regions, and filters. Therefore, ANOVA was performed for each implant material and showed a significant interaction between tooth region and filter in both titanium (*P* < 0.001) and zirconium dioxide (*P* < 0.001) implants, making it impossible to interpret the effects of tooth region and filters.

It was also necessary to build a model with implant material and tooth regions, this time comparing filters to each other within each combination of implant and tooth. The results are shown in Table 2. The lowest values of quantification of artifacts in titanium implants were comparable to those in zirconium dioxide ones when the same implant sites were directly compared to each other for the two implant materials.

As for the filters used, the lowest values of quantification of artifacts for both implant materials were found with BAR and Multi-CDT NR filters. The values related to BAR filter were the lowest, regardless of the site of implant. In the case of titanium implants, it was also mentioned that the BAR filter differed statistically from all other ones, including the Multi-CDT NR filter, except in the regions of teeth #43 and #44. However, no statistical difference between BAR and Multi-CDT NR filters was observed. For zirconium dioxide implants, there was a statistically significant difference between BAR filter (with better results) and all other filters.

It was possible to observe the highest values of quantification of artifacts in the filters belonging to the OnDemand software (i.e., Filter 1x and Filter 2x). Both these filters had no statistical differences between them, except in the region of tooth #46 for titanium implants. As for the titanium implants, in the regions of teeth #44 and #46, and zirconium dioxide implants, in the region of tooth #44, the values of quantification of artifacts in images filtered with Filter 1x were higher than those in unfiltered ones, but they did not differ statistically from each other.

## 4. Discussion

The importance of this study resides in the fact that CBCT images are frequently needed in the dental practice, and thus, the radiologist is faced with artifacts in images of implants previously placed in regions close to the sites to be analyzed. These artifacts can impair the clarity of information as they can be confounded with dental fractures and hypodense regions, leading to erroneous interpretations and inaccurate diagnosis [2].

There are a couple of optimization algorithms which can be applied to CBCT images for reduction of metal artifact and noise reduction in order to improve the image quality during image reconstruction. In this context, the use of postprocessing filters can minimize this process and favor diagnostic accuracy, which is essential in the practice of radiology. There are many software and resources available to quantify their capacity to improve the quality of images by reducing the amount of artifacts. In addition, the effects of filters should be better analyzed in relation to the region of the mandible, although this issue had already been addressed elsewhere [1, 26].

With regard to the region of the teeth where the implants were placed, it is known that the grayscale values are not uniform in all regions. In fact, the comparison of the quantification of metal artifacts between regions indicates that there is a greater tendency for an increase in the region of incisors [14, 27]. However, it is interesting to note that the lowest values were observed for zirconium dioxide implants inserted in the region of incisors, since this type of implant is more aesthetically justified because certain metal implants (e.g., titanium) could have poor results.

In this study, the results showed that the highest values of quantification of artifacts occurred in the regions of teeth #41 and #43 for both titanium and zirconium dioxide implants, whereas the lowest values occurred in the posterior regions, particularly in the region of tooth #46. These results corroborate those reported by Machado et al. [14], who found higher values of quantification of artifacts in implants placed in the anterior regions. It should be also emphasized that the cervical region is the most affected by artifacts in axial images, which justifies our choice for axial scans at 3.0 mm below the implant base. Also, as for the influence of tooth region on dental arches and artifacts, a study by Fontenele et al. [28] reported that the posterior region of the mandible had lower grayscale values than the anterior regions. However, the study assessed artifacts produced by intracanal metal retainers made of different materials.

With regard to the effect of the material of the implants used in our study (i.e., titanium and zirconium dioxide) and artifacts produced by them, it was shown that the highest values of quantification of artifacts were observed in zirconium dioxide implants compared to titanium ones. Shahmirzadi et al. [29] evaluated artifacts generated by dental implants in CBCT images by using three MAR algorithm conditions (i.e., preacquisition MAR, postacquisition MAR, and no MAR) and two peak kilovoltage (84 and 90 kVp) settings. Analysis of all three MAR conditions showed that there were substantially more severe artifacts when no MAR algorithm was used compared to either of the two MAR algorithm conditions for dental implant materials. It was also shown that artifacts can be minimized by using a titanium-zirconium alloy at a 90 kVp setting in both MAR conditions [29].

The results are in line with those of previous studies, such as that by Vasconcelos et al. [30], who evaluated different acquisition protocols and their effect on the quality of images of titanium and zirconium dioxide implants. These authors concluded that zirconium dioxide implants produced more artifacts than the titanium ones, highlighting that an increase in kilovoltage reduces artifact formation with both implants materials. In our study, such a parameter was not considered as the main objective was to assess the effect of filters (OnDemand and e-Vol DX software) on the quantification of these artifacts. Variation of acquisition parameters (e.g., spatial resolution), combined with the effect of these filters on artifacts, should be further studied after analysis of the initial results of the filters.

Other studies [2, 31, 32] also corroborate our results, in which the highest values of quantification of artifacts in CBCT images occurred with zirconium dioxide implants compared to the titanium ones.

The results showed that, among the filters provided by software, BAR and Multi-CDT NR filters promoted greater reduction in the quantification of artifacts, whereas Filters 1x and 2x had mean values very similar to those of unfiltered images, often with no significant differences between them. In other words, unfiltered images and those filtered with OnDemand filters had similar results in relation to the artifacts produced. Emphasis is given to the region of tooth #41 with titanium implant, region of tooth #44 with both titanium and zirconium dioxide implants, and region of tooth #46 with zirconium dioxide implant.

When BAR filter was analyzed in relation to titanium implants, there was a significant reduction in the mean values of quantification of artifacts, ranging from 79.6% (region of tooth #46) to 68.3% (region of tooth #44) in unfiltered images.

In the case of zirconium dioxide implants and BAR filter, the mean values of quantification of artifacts ranged from 78.2% (region of #43) to 68.3% (region of tooth #41) in unfiltered images. The mean values of quantification of artifacts in images filtered with BAR filter differed statistically from those using all others regarding all regions, which further highlights the effect of the other filters evaluated. This finding seems to be interesting as zirconium dioxide implants showed higher values of quantification of artifacts than those of the titanium ones, which could reflect in an improvement of the images. This study did not assess the subjective quality of the images, which is in fact a limitation of our results. This means that it would be valid to combine these quantitative findings with other evaluations, although such objectives are beyond the scope of this study.

This is the first study to use filters supplied by e-Vol DX software to objectively assess the reduction of metal artifacts in images of dental implants. Overall, one can observe that there was a reduction in the values of artifact quantification when BAR filter was used, regardless of the implant material, but dependent on the region of placement. There are a few studies comparing postprocessing filters available in software in relation to the quantification of artifacts generated by implants. Some of these studies compared only the use of these filters for detection of fractures in teeth with intracanal metal posts [12, 33], but none evaluated filters supplied by e-Vol DX software, probably because they are a relatively new product.

A study by Gregoris Rabelo et al. [22] verified the effectiveness of using the BAR filter for reducing artifacts caused by intracanal metal posts in CBCT images. The authors emphasized that this finding opens a new possibility for clinical applications of this filter. However, unfortunately, very few studies have been carried out with the aim of investigating the efficacy of BAR filter in reducing artifacts produced by high-density materials, which makes comparison difficult. Therefore, our results, which are in agreement with those of the aforementioned study, strengthen the arguments that the use of this filter is effective.

These study results need to be interpreted considering some limitations. The phantoms do not have soft tissue simulation and may not represent the natural structures of the periodontal composition. We used a single CBCT scanner with only one protocol. Future studies should be conducted with better simulation methods, which could be more in accordance with the living being and daily practice in several radiological centers.

Our study focused on the use of enhancement filters for possible reduction of artifacts, specifically those arising from dental implants. The use of tools such as MAR, activated at the time of image acquisition, is already well evidenced in the literature in terms of effectiveness. However, in systems such as the ones used here, which do not have this tool, the use of manipulation resources (i.e., filters) by software has already been recommended to improve the quality of images, but always taking into account adequacy of voxel (for reduction in noise) and protocols related to energy factors (i.e., kV and mAs). It is important to emphasize that the results of our *in vitro* study were obtained under controlled conditions and therefore without secondary influences. Thus, there is a need for future studies on various diagnostic tasks under clinical conditions.

## 5. Conclusion

The BAR filter of the e-Vol DX software proved to be effective in reducing the quantification of artifacts generated by dental implants, and its action can be influenced depending on the position of the implant in the dental arch.

## Figures and Tables

**Table 1:** Quantification mean (standard deviation) values of the artifact with titanium and zirconium dioxide implants.

Titanium implant			Zirconium dioxide		
Filter	Mean	SD	Filter	Mean	SD
43			43		
Filter 1x	6.4	0.3	Filter 1x	9.2	0.0
Filter 2x	6.3	0.2	Filter 2x	9.1	0.1
Filter BAR	5.7	0.0	Filter BAR	7.4	0.1
Multi-CDT NR	6.0	0.0	Multi-CDT NR	8.4	0.1
Nonfiltered	6.7	0.0	Nonfiltered	9.5	0.0
41			41		
Filter 1x	6.6	0.1	Filter 1x	9.3	0.2
Filter 2x	6.5	0.2	Filter 2x	9.2	0.0
Filter BAR	5.7	0.1	Filter BAR	6.7	0.3
Multi-CDT NR	6.3	0.1	Multi-CDT NR	7.6	0.0
Nonfiltered	6.9	0.2	Nonfiltered	9.8	0.1
44			44		
Filter 1x	6.6	0.2	Filter 1x	9.2	0.2
Filter 2x	6.5	0.2	Filter 2x	8.9	0.2
Filter BAR	5.1	0.0	Filter BAR	7.2	0.1
Multi-CDT NR	5.4	0.1	Multi-CDT NR	8.4	0.2
Nonfiltered	6.5	0.1	Nonfiltered	9.1	0.0
46			46		
Filter 1x	6.4	0.3	Filter 1x	8.9	0.0
Filter 2x	6.0	0.2	Filter 2x	8.8	0.0
Filter BAR	5.1	0.1	Filter BAR	6.6	0.2
Multi-CDT NR	5.9	0.2	Multi-CDT NR	7.2	0.1
Nonfiltered	6.2	0.2	Nonfiltered	9.1	0.2

*N*: number of scans; SD: standard deviation.

**Table 2:** Comparison between filters/teeth for titanium and zirconium dioxide implant.

Titanium implant				Zirconium dioxide			
Filter	Median	Result^∗^	SD	Filter	Median	Result^∗^	SD
41				41			
BAR	7.4	C	0.1	BAR	6.7	D	0.3
Multi-CDT NR	8.4	B	0.1	Multi-CDT NR	7.6	C	0.0
2x	9.1	AB	0.2	2x	9.2	B	0.0
1x	9.2	AB	0.1	1x	9.3	B	0.2
Nonfiltered	9.5	A	0.2	Nonfiltered	9.8	A	0.1
43				43			
BAR	5.7	D	0.0	BAR	7.4	D	0.1
Multi-CDT NR	6.0	CD	0.0	Multi-CDT NR	8.4	C	0.1
2x	6.3	BC	0.2	2x	9.1	B	0.1
1x	6.4	AB	0.3	1x	9.2	AB	0.0
Nonfiltered	6.7	A	0.0	Nonfiltered	9.5	A	0.0
44				44			
BAR	6.7	B	0.0	BAR	7.2	C	0.1
Multi-CDT NR	7.6	B	0.1	Multi-CDT NR	8.4	B	0.2
2x	9.2	A	0.2	2x	8.9	A	0.2
Nonfiltered	9.3	A	0.1	Nonfiltered	9.1	A	0.0
1x	9.8	A	0.2	1x	9.2	A	0.2
46				46			
BAR	5.1	D	0.1	BAR	6.6	C	0.2
Multi-CDT NR	5.9	C	0.1	Multi-CDT NR	7.2	B	0.1
2x	6.0	BC	0.2	2x	8.8	A	0.0
Nonfiltered	6.2	AB	0.1	1x	8.9	A	0.0
1x	6.4	A	0.3	Nonfiltered	9.1	A	0.2

^∗^Medians followed by the same letters do not differ significantly from each other.

## Data Availability

The data used to support the findings of this study are available to interested readers upon reasonable request.
